# Central motor control failure in fibromyalgia: a surface electromyography study

**DOI:** 10.1186/1471-2474-10-78

**Published:** 2009-07-01

**Authors:** Roberto Casale, Piercarlo Sarzi-Puttini, Fabiola Atzeni, Marco Gazzoni, Dan Buskila, Alberto Rainoldi

**Affiliations:** 1 Department of Clinical Neurophysiology and Pain Rehabilitation Unit, "Salvatore Maugeri" Foundation, IRCCS, Scientific Institute of Montescano, Montescano (PV), Italy; 2 Rheumatology Unit, Luigi Sacco University Hospital, Milan, Italy; 3 Laboratory for Neuromuscular System Engineering (LISiN), Department of Electronics, Polytechnic of Turin, Turin, Italy; 4 Department of Internal Medicine H, Soroka Medical Center, Ben Gurion University, BeerSheva, Israel; 5 Motor Science Research Centre, University School of Motor and Sport Sciences (SUISM), University of Turin, Turin, Italy

## Abstract

**Background:**

Fibromyalgia (FM) is characterised by diffuse musculoskeletal pain and stiffness at multiple sites, tender points in characteristic locations, and the frequent presence of symptoms such as fatigue. The aim of this study was to assess whether the myoelectrical manifestations of fatigue in patients affected by FM are central or peripheral in origin.

**Methods:**

Eight female patients aged 55.6 ± 13.6 years (FM group) and eight healthy female volunteers aged 50.3 ± 9.3 years (MCG) were studied by means of non-invasive surface electromyography (s-EMG) involving a linear array of 16 electrodes placed on the skin overlying the biceps brachii muscle, with muscle fatigue being evoked by means of voluntary and involuntary (electrically elicited) contractions. Maximal voluntary contractions (MVCs), motor unit action potential conduction velocity distributions (mean ± SD and skewness), and the mean power frequency of the spectrum (MNF) were estimated in order to assess whether there were any significant differences between the two groups and contraction types.

**Results:**

The motor pattern of recruitment during voluntary contractions was altered in the FM patients, who also showed fewer myoelectrical manifestations of fatigue (normalised conduction velocity rate of changes: -0.074 ± 0.052%/s in FM vs -0.196 ± 0.133%/s in MCG; normalised MNF rate of changes: -0.29 ± 0.16%/s in FM vs -0.66 ± 0.34%/s in MCG). Mean conduction velocity distribution and skewnesses values were higher (p < 0.01) in the FM group. There were no between-group differences in the results obtained from the electrically elicited contractions.

**Conclusion:**

The apparent paradox of fewer myoelectrical manifestations of fatigue in FM is the electrophysiological expression of muscle remodelling in terms of the prevalence of slow conducting fatigue-resistant type I fibres. As the only between-group differences concerned voluntary contractions, they are probably more related to central motor control failure than muscle membrane alterations, which suggests pathological muscle fibre remodelling related to altered suprasegmental control.

## Background

FM is characterised by diffuse musculoskeletal pain and stiffness at multiple sites, tender points at characteristic locations, and the frequent presence of symptoms such as fatigue, poor sleep, irritable bowel symptoms and chronic headache [1,2].

Various theories have been put forward to explain such a wide range of symptoms, including altered nociceptive afferent input from muscles with the sensitisation of primary afferent pathways [3], systemic failure of the sensory integration of nociceptive inputs and anti-nociceptive responses [4,5], primitive sleep-related electroencephalogram (EEG) alterations [6], and alterations in the neuroendocrine and immune systems [7]. A review of the various theories has recently been published [8]. Studies of the main features of FM (muscle pain and fatigue) have failed to produce unequivocal results in terms of functional muscle changes as a possible pathological basis for the presence of muscle pain, and the results of biopsy studies do not support any clear-cut conclusions as some [9,10] have failed to find any definite evidence of muscle disease, and others have only found non-specific myopathological patterns [11]. Furthermore, the traditional invasive approach is not well tolerated, cannot be frequently repeated, and small bioptic specimens can only provide a limited description of the sampled muscle. It also leaves a number of open questions concerning the reproducibility of the results and the possibility of inferring the changes induced by treatments.

Multichannel surface electromyography (s-EMG) signal acquisition and processing can extract information from the signal redundancy provided by the multichannel recording [12-14] that correlates with *acute *alterations in motor unit recruitment strategies [15] and/or *chronic *modifications in the type, distribution, number or size of muscle fibres [16], and is reflected by alterations in mechanical and/or s-EMG manifestations of fatigue.

Although some previously published studies have related FM and s-EMG [17,18], they provide no information concerning changes in the myoelectrical manifestations of fatigue as a marker of muscle functional reorganisation in FM. The aims of this non-invasive EMG-based (s-EMG) study of fibromyalgic patients and a matched control group were to assess whether FM muscle is different in terms of the development of muscle fatigue (i.e. whether there are any differences in the variables estimated from s-EMG signals), and whether these differences (if any) are associated with pathological alteration in *central *sensory-motor control strategies or *peripheral *changes in the neuromuscular system.

## Methods

### Subjects

The study involved 16 women: eight had been diagnosed as having fibromyalgia on the basis of the American College of Rheumatology (ACR) criteria [1] (the FM group); the other eight were healthy sedentary controls matched for age, gender and physical activity [19] (MCG). Pain was assessed using the McGill Pain Questionnaire Short Form (MPQ-SF) [20] and the severity of FM symptoms by means of the FibroFatigue Scale (FFS) [21]. (Table 1).

**Table 1:** Subject characteristics

	MCG	FM	p level
**Age **(years)	50.3 ± 9.3.	55.6 ± 13.6	NS
**Body Mass **(kg)	59.4 ± 18.3	56.25 ± 16	NS
**Height **(cm)	162.5 ± 10.3	159.3 ± 6.5	NS
**Physical activity **[18]	4.9 ± 1.9	3.3 ± 1.5	NS
**Symptoms duration **(years)		9.3 ± 1.3	
**MPQ-SF **[19]			
Intensity (0–45)		26.1 ± 9.9	
Total words (0–15)		11.1 ± 4.3	
Present Pain Intensity (0–5 pts scale)		3.4 ± 0.5	
**FFS **[20]			
Total score		39.1 ± 2.4	

Values are expressed as Mean ± SD.

FM = Fibromyalgic Patients; MCG = Matched Control Group; MPQ-SF = McGill Pain Questionnaire Short Form; FFS = Fibro Fatigue Score; NS = non significative

The possible non-homogeneity of the FM population has been reported since the early nineties [22,23], but the subgroup distribution of the whole FM population has not yet been fully studied. Possible differentiations have been related to the onset of the disease: reactive traumatic (both physical and emotional) or primitive, but there are no substantial clinical differences between the two groups [24]. This has also been also confirmed by more recent data from Riberto et al. [25]. For these reasons the sample was considered clinically homogeneous and subjects were only excluded if they were simultaneously affected by any other rheumatological disease or high blood pressure (or receiving anti-hypertensive treatment such as clonidine); any infectious or viral diseases during the previous three months; metabolic or endocrine diseases; neuromuscular diseases or any other neurological or psychiatric disorder that may have hindered them from doing the required task; or if they were regularly receiving antidepressants, opiates or anti-epileptic drugs. All of the enrolled subjects underwent 48 hours' washout of analgesic drugs. Although paracetamol was allowed as a rescue drug, none of the subjects were under the influence of any drug during the s-EMG recording session.

The thickness of the subcutaneous tissue under the electrodes was estimated using a skin fold caliper (Gima, Gessate, Milan, Italy) in order to eliminate the volume conductor between the signal source (the muscle) and the recording device (the electrodes) as a confounding factor between the groups [26].

The study was approved by the "Foundation Salvatore Maugeri", Pavia Italy, Ethics Committee, and was conducted in accordance with the principles of the Declaration of Helsinki; all of the subjects gave their informed consent.

### Instrumental measurements

The myoelectrical signals were detected from the biceps brachii muscle of the dominant side, which was selected because: 1) it is a muscle, and not a site of any of the 18 myalgic points used to classify FM, and was not a site of spontaneous pain in our patients, and so the presence of incident pain could not be considered the local cause of an altered muscle contraction; 2) it is easily accessible for electrode placement; 3) it does not cause patient discomfort; 4) it allows the recording of high quality signals; and 5) published normative data are available [27].

After being picked up by a linear array of 16 electrodes (1 mm diameter silver contacts positioned 10 mm apart) in single differential configuration [28], the myoelectrical signals were passed through a 10–450 Hz bandwidth filter, amplified (EMG16-16 channel amplifier, LISiN Bioengineering Centre, Turin Polytechnic, Italy), sampled at 2048 Hz, digitised by a 12-bit A/D converter (DAQCARD-6024E National Instruments, Austin, Texas, USA), and stored on a personal computer.

### Experimental protocol

**Figure 1:**
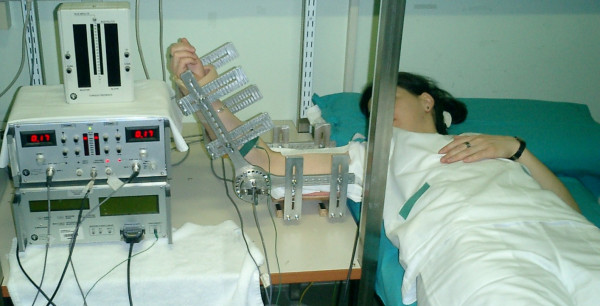
**The experimental set up**. The subject lies supine on a bed with the dominant arm horizontal and abducted to 90°. The forearm is 120° flexed (180° being full extension), with the hand supinated. The arm is placed in an isometric brace equipped with two torque transducers (one on each side of the arm) connected to a display that provides the subject with visual feedback of the torque level produced. The myoelectrical signals are picked up by a linear array of 16 electrodes placed on the skin overlying the biceps brachii muscle. The linear array can be identified as a white band on the biceps brachii.

Each subject lay supine on a bed with the dominant arm horizontal and abducted to 90°. The forearm was 120° flexed (180° being full extension), with the hand supinated. The arm was placed in an isometric brace (MISO1, LISiN Bioengineering Centre, Turin Polytechnic, Italy), which was equipped with two torque transducers (one on each side of the arm) and connected to a display that provided the subject with visual feedback of the torque level produced (Figure 1).

The subjects were asked to perform a brief (3–5 second) isometric flexion contraction that allowed the quality of the myoelectrical signals to match the criteria described in detail by Rainoldi et al. [29], and then performed three 3-second maximum voluntary contractions (MVCs) under isometric conditions, separated by 5-minute intervals. The contraction showing the highest force value was selected as the reference MVC, thus allowing sub-maximal targets to be set on the visual feedback display.

After the MVC had been assessed, the motor points of the biceps brachii were identified and marked on the skin, and the one with the greatest mechanical response and lowest current intensity was chosen for the electrically elicited contraction session.

After placing an adhesive stimulation electrode (area = 9 cm^2^, Spes Medica, Vignate, Milan, Italy) on the selected motor point, a rectangular current pulse was applied using a time width of 0.3 ms and a frequency of 25 Hz; the stimulation was supramaximal (about 10% above the level generating the peak M-wave or the maximum level tolerated by the subjects). Two electrically elicited 30-second contractions separated by a 10-minute rest were performed at each experimental session. The 25 Hz stimulation was judged not to be unbearable or uncomfortable by all of the subjects in both groups.

After a further five minutes' rest, the subjects were asked to perform two voluntary contractions at 30% MVC and two at 60% MVC in a randomised sequence; the contractions lasted 30 seconds and were separated by 5-minute rest periods. This procedure is usually adopted in our lab to allow subjects to familiarise themselves with the protocol without inducing fatigue or a learning effect; only the data relating to the second contractionswere recorded and analysed.

### Data analysis

The initial values and rates of change in mean spectral frequency (MNF), average rectified value (ARV) and conduction velocity (CV) were computed off-line by means of numerical algorithms [30] using non-overlapping signal epochs of 0.5 seconds, thus generating 60 estimates of each variable during the 30-second contractions.

The correlation coefficient (CC) between the two adjacent double differential (DD) signals was used to ensure correct electrode positioning and the reliability of the CV estimates.

Linear regression analysis was used to calculate the initial values and rates of change in MNF, ARV and CV during the voluntary and electrically elicited contractions. The normalised rate of change (the ratio between the rate of change and the initial value) was also calculated for all variables. As demonstrated elsewhere [27], this normalisation highlights differences in the myoelectrical manifestations of fatigue that may be related to different pools of activated motor units.

Motor unit action potentials (MUAPs) were extracted from the 13 DD channels only during the voluntary contraction [13]. For each identified MUAP, the muscle fibre CV was estimated from the surface EMG signal using 3–7 adjacent DD channels in order to obtain a CC of > 0.75 and a CV > 8 m/s [31]. The selected channels included those used to estimate the global EMG variables.

The MUAP CV distributions were calculated for each contraction using epochs of one second. In this way, the initial values and rates of change were obtained by pooling the behaviour of each MU, and not by using the whole signal providing the "global" variables.

In order to compare general behaviour in the MCG and FM group, the data were pooled at each contraction level.

The non-parametric Mann-Whitney U test for independent samples was used to identify any significant between-group differences in the s-EMG variables, and the non-parametric Kolmogorov-Smirnov test was used to compare CV distributions. A p value of < 0.05 was considered statistically significant.

## Results

None of the subjects reported any localised biceps brachii muscle pain during the MVCs or any worsening in FM pain interfering with their ability to perform the requested task, possibly because of the brevity of the required muscle contraction (3 sec).

Table 2 shows the estimates of the EMG variables during the electrically elicited contractions. There were no between-group differences in the initial values or rates of change. The 25 Hz stimulation was judged to be not unbearable or uncomfortable by all of the subjects in both groups.

**Table 2:** The results of the non-parametric Mann-Whitney U test

	MCG	FM	p level
CV initial values (m/s)	4.07 ± 0.45	4.32 ± 0.36	NS
CV rate of change (m/s^2^)	-0.02 ± 0.01	-0.02 ± 0.01	NS
CV norm. rate of change (%/s)	-0.50 ± 0.29	-0.46 ± 0.23	NS
MNF initial values (Hz)	62.28 ± 17.22	57.63 ± 13.26	NS
MNF rate of change (Hz/s)	-0.43 ± 0.29	-0.44 ± 0.19	NS
MNF norm. rate of change (%/s)	-0.64 ± 0.31	-0.74 ± 0.20	NS
ARV initial values (μV)	28.53 ± 13.12	21.24 ± 9.44	NS
ARV rate of change (μV/s)	0.17 ± 0.22	0.16 ± 0.15	NS
ARV norm. rate of change (%/s)	0.58 ± 0.72	0.78 ± 0.64	NS

*Mean *values ( ± SD) of EMG variables during electrically elicited contractions in the two groups. The results of the non parametric Mann-Whitney U test are reported.

MCG = Matched Control Group, FM = Fibromyalgic group, MVC = maximal voluntary contraction, CV = conduction velocity, MNF = mean spectral frequency, ARV = average rectified value; NS = non significative

There were no statistically significant differences in the voluntary contractions at 30% MVC.

Table 3 shows the s-EMG variables during voluntary contractions at 60% of MVC and maximal MVC. The MVCs in the two groups were not statistically different. There were no between-group differences in the initial values and rates of change of the EMG variables, except for the MNF rate of change and the normalized rate of change at 60% MVC, which were higher in the MCG, thus indicating greater myoelectrical fatigue.

**Table 3:** Mean values ( ± SD) of mechanical and EMG variables during voluntary contractions at 60%

	MCG	FM	p level
MVC (Nm)	18.16 ± 7.89	14.97 ± 6.73	NS
CV initial values (m/s)	4.37 ± 0.34	4.58 ± 0.49	NS
CV rate of change (m/s^2^)	-0.01 ± 0.01	-0.01 ± 0.01	NS
CV norm. rate of change (%/s)	-0.25 ± 0.33	-0.19 ± 0.19	NS
MNF initial values (Hz)	93.00 ± 14.58	87.21 ± 25.35	NS
MNF rate of change (Hz/s)	**-0.59 ± 0.29**	**-0.25 ± 0.14**	**0.036**
MNF norm. rate of change (%/s)	**-0.66 ± 0.34**	**-0.29 ± 0.16**	**0.046**
ARV initial values (μV)	93.40 ± 46.03	77.84 ± 54.02	NS
ARV rate of change (μV/s)	1.29 ± 1.47	0.68 ± 0.66	NS
ARV norm. rate of change (%/s)	1.23 ± 1.24	0.74 ± 0.41	NS

Mean values ( ± SD) of mechanical and EMG variables during voluntary contractions at 60% MVC in the two groups. The results of the non parametric Mann-Whitney U test (in bold those significant) are reported.

MCG = Matched Control Group, FM = Fibromyalgic Group, MVC = maximal voluntary contraction, CV = conduction velocity, MNF = mean spectral frequency, ARV = average rectified value; NS = non significative

The CV rate of change and the normalised rate of change calculated from the extracted MUAP pool at 60% MVC were both higher in the MCG (-0.009 ± 0.007 *vs *-0.004 ± 0.003 m/s^2^, p = 0.031; and -0.196 ± 0.133%/s *vs *-0.074 ± 0.052%/s, p = 0.015), and in line with those obtained from the global MNF estimates.

**Figure 2:**
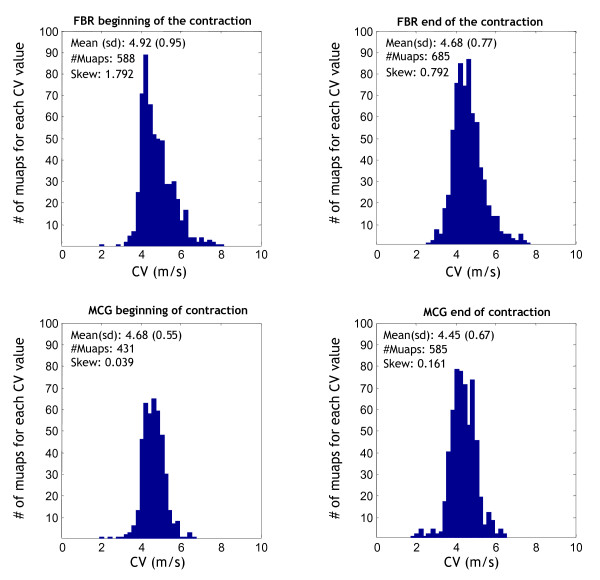
**CV distributions in the FM and MCG groups: beginning and ending at 60% MVC**. CV distributions in the FM (top) and MCG group (bottom) shown as two-second epochs at the beginning and end of a 60% MVC. Mean CV distribution values and skewnesses were significantly higher in the FM group (p < 0.001, Kolmogorov-Smirnov test). The numbers of identified MUAPs are also shown. MCG = matched control group; FM = fibromyalgia; s-EMG = surface electromyography; MVC = maximal voluntary contraction; MNF = mean power frequency of the spectrum; CV = conduction velocity; MPQ-SF = McGill Pain Questionnaire Short Form; FFS = Fibrofatigue Scale; ARV = average rectified value; CC = correlation coefficient; MUAP = motor unit action potential; MU = motor unit; DD = double differential.

CV distributions were statistically *broader *in the FM group at the beginning and end of the 60% MVC (p < 0.001, Kolmogorov-Smirnov test, Figure 2).

The CV distribution symmetry index (skewness) of the two groups was higher in the FM group at 60% MVC (1.77 ± 1.29 *vs *0.09 ± 0.27; p < 0.00001 Mann Whitney U test). Positive values correspond to a deviation from the normal bell-shaped curve, and show that the CV distribution curves in the symptomatic group were steeper on the left because of the contribution of higher CV values. This behaviour was more pronounced at the beginning of the contraction.

The absence of any significant between-group difference in subcutaneous tissue thickness means that this did not have a confounding effect on the MNF or CV estimates [26].

## Discussion

The correlation between muscle pain and the sensation of fatigue in FM is considered a generalised muscle response [32], and has been studied using traditional EMG needle electrodes [18] and the surface montages mainly used for s-EMG biofeedback [32]. The latter have also been used for therapeutical interventions although, as stated in a recent overview of rehabilitation interventions in FM [33], their efficacy is still unproven. However, neither technique has detected any alterations in the muscle contractions of FM patients. Furthermore, they cannot be used to investigate the rate of the development of fatigue in different muscle fibres or possible differences in the fibre constitution of normal and FM muscles as they do not provide clear-cut data concerning primary muscle involvement in the abnormal perception of fatigue characteristic of FM.

The perception of fatigue and its mechanical consequences (the impossibility of maintaining a given motor task) are the final expression of a series of electrochemical events that start within the neuromuscular system and go from the beginning of the motor command to the end of the muscle contraction, and its has been clearly documented that FM patients complain about fatigue and misjudge their physical activity [32,34]. This biases almost all of the studies based on prolonged, strenuous or sustained periods of muscle contraction or the use of needle electrode because FM patient have very low pain thresholds, but can be avoided by analysing s-EMG recordings of short (3-second) muscle contraction periods which, at least in our experience, do not give rise to any idiosyncrasies or kinesiophobic behaviour.

Changes in s-EMG signals over time reflect the early physiological phenomena evolving during sustained muscle contraction, and account for the so-called "myoelectrical manifestations of fatigue", which are affected by (and therefore reflect) the constitution of muscle fibres. One way of distinguishing the two main types of motor unit (MU) is by means of their susceptibility to electrical fatigue, and their mechanical response to it: fast (type II) MUs produce force twitches characterised by a short time to peak and rapid fatigue, whereas slow (type I) MUs take longer to peak and ans are slower to tire. The former usually have larger fibres and higher CV values than the latter [12]. In other words, a muscle with more type I fibres develops less myoelectrical fatigue, whereas one characterised by a high percentage of type II fibres is more powerful but less resistant to fatigue.

The strategy of activating these two main types of MU during a voluntary contraction is based on Henneman's "size principle", which states that progressively larger MUs characterised by larger CVs are recruited with increasing muscle force [35]. In practical term, a 30% MVC does not activate the whole MU pool (mainly type I fibres), whereas an MVC of 60% or more does (type I and II fibres) [36-38].

The absence of significant differences between the two groups in the s-EMG parameters observed during 30% MVC contractions was due to the partial activation of the MU pool induced at submaximal contraction level. This is in line with the significant findings observed at 60% MVC: i.e. a contraction level at which the whole biceps brachii MU pool is recruited.

MU recruitment can be modified by diseases, aging and/or processes of adaptation: for example, the physiological reduction in the number and size of type II fibers in the elderly leads to paradoxically fewer myoelectrical manifestations of fatigue than in younger subjects [39].

On the basis of these premises, each set of data will be discussed separately.

### Maximal voluntary contractions (MVCs)

Functional changes in FM muscles are often assessed by measuring strength and endurance, and the most frequent findings are reduced exercise endurance [32], reduced voluntary muscle force [6,7], and a perceived greater effort during fatigue [32]. However, other studies have found no differences in maximal voluntary strength between FM and healthy subjects [16,33], and their results are supported by our own MVC findings, which suggest that no strong and unequivocal muscle fibre modifications (in distribution, size or type) occur in FM (Table 2).

### Myoelectrical manifestations of fatigue

Using both voluntary and electrically elicited contractions makes it possible to identify the site of impairment along the neuromuscular chain: differences in the myoelectric manifestations of fatigue observed only during voluntary contractions can be related to an altered motor control strategy, whereas differences observed only during electrically elicited contractions can be considered as being mainly due to membrane properties [15].

As we found between-group differences in the EMG variables assessed on the basis of signals recorded during voluntary but not electrically elicited contractions, it is possible to localise functional impairment in FM at a higher level than that of the muscle membrane.

In line with other studies [18,40-43], although adopting a different approach, we can conclude that the lack of myoelectrical fatigue and the associated muscle symptoms in FM syndrome are more likely to be generated by an alteration in central nervous system motor recruitment strategies than by peripheral mechanisms within the muscle itself [43,44]. The reduced EMG manifestations of fatigue and the type II fibre hypotrophy shown histologically in FM [10,45-49]. have also been found in chronic fatigue syndrome and interpreted as a sign of de-conditioning [50].

The CV rate of change and the normalised rate of change estimated from the extracted MUAP pool were in line with the MNF findings. As CV estimates provide a clear physiological description of the fatiguing mechanism, these results confirm the MNF findings.

### Distribution of muscle fibre conduction velocity (CV)

Differences in the distribution and skewness of CV are usually explained as a consequence of different MU pool recruitment, with increases in average CV being due to an increase in faster MU recruitment [51]. The mean CV distribution and skewness values in our FM patients were higher than those in the MCG during the 60% MVC, which leads us to conclude that FM patients recruit more of the faster MUs, and so an increase in the s-EMG signs of muscle fatigue can be expected. Similar observations were made by Gerdle et al. in their study of trapezius muscle [52]: the CV values in the FM subjects were higher than those in the controls during low level contractions (no load, 1 kg, and 2 kg).

The dissociation between the curve of CV distribution and the myoelectrical manifestations of fatigue in our FM patients can be interpreted on the basis of an anomalous sensory-motor system pattern and the use of non-physiological strategies in managing functional motor tasks, as seen in chronic fatigue syndrome [53].

The CV distribution curves and estimates from each extracted MUAP offer two not necessarily matching descriptions because fast MUAPs can be recruited frequently (thus increasing the number of bins on the right side of the distribution: see Figure 2, upper row) but only for short periods without increasing the myoelectrical manifestations of fatigue (see Table 2).

### Altered order of recruitment in FM

The theoretical possibility of an altered order of recruitment/de-recruitment has recently been experimentally demonstrated by means of a technique based on EMG signals [54] and, given the findings described above, it can be speculated that patients affected by FM use compensatory motor strategies to recruit MUs in a different manner from that expected predicted by Henneman's principle. The wider CV distributions and fewer myoelectrical manifestations of fatigue observed in our FM group may therefore be due to the recruitment/de-recruitment of a partially or non-fatigued MU.

In an attempt to confirm this speculative explanation, the coefficient of variation (COV) of the force generated during isometric exercise at 60% MVC was calculated with the assumption that the hypothesised higher level of recruitment/de-recruitment in the FM group is related to a higher COV in mechanical output. The COVs in the two groups were not statistically different, but did show a trend in the hypothesised direction.

An altered recruitment/de-recruitment pattern can be considered a motor response to an alteration in the central integration of sensory inputs [51,55], and the presence of central hyper-excitability has also been observed by others as a neurophysiological mechanism of muscle pain in FM [5,40,51,56] Our findings confirm the concomitant presence of central sensitisation and functional abnormalities in muscle contraction (i.e. the recruitment and de-recruitment of muscle fibres during a 60% MVC) in FM during maximal/sub-maximal load and static contraction observed by other authors [57].

## Conclusion

The observed alteration in physiological recruitment order seems to be the main electrophysiological characteristic of FM central motor control failure, and can be considered a sort of kinesiophobic defensive/compensatory strategy [58].

Our findings suggest a strong correlation between the established presence of altered sensory afferents and integration, and the concomitant presence of alterations in the efferent central motor command revealed by the use of non-physiological strategies in managing functional motor tasks. As far as we know this relationship has never previously been reported and supported by s-EMG data.

## Competing interests

The authors declare that they have no competing interests.

## Authors' contributions

RC: study coordination, study design, s-EMG recording, s-EMG analysis, manuscript drawing; PSP-FA: study design, patients selection and enrollement, manuscript drawing; DB: study design, manuscript drawing; MG-AR: study design (s-EMG recording and analysis), s-EMG analysis, statistical analysis, manuscript drawing All authors read and approved the final manuscript.

## Pre-publication history

The pre-publication history for this paper can be accessed here:


